# MoSAIC: An Integrated and Modular Workflow for Confident Analysis of Protein Post-Translational Modification Landscapes

**DOI:** 10.1016/j.mcpro.2025.101502

**Published:** 2025-12-24

**Authors:** Yuanwei Xu, Lijun Chen, T. Mamie Lih, Yingwei Hu, Hui Zhang

**Affiliations:** 1 Department of Pathology, Johns Hopkins University School of Medicine, Baltimore, Maryland, USA; 2 Department of Chemical and Biomolecular Engineering, Johns Hopkins University, Baltimore, Maryland, USA

**Keywords:** mass spectrometry, post-translational modifications, data-independent acquisition, data-dependent acquisition, tandem mass tag, phosphorylation, glycosylation, acetylation, ubiquitination, proteomics workflow, PTM crosstalk

## Abstract

Investigating multiple protein post-translational modifications (PTMs) is critical for unraveling the complexities of protein regulation and the dynamic interplay among PTMs, a growing focus in proteomics. However, simultaneous analysis of diverse PTMs remains a significant technical challenge, as existing workflows struggle to balance throughput, sensitivity, and reproducibility, particularly when sample amounts are limited. To address these limitations, we present MoSAIC, a multi-PTM workflow integrating coenrichment strategies, multiplexing, fractionation, hybrid data acquisition, and unified data analysis, optimized for clinically relevant biological samples. This approach targets phosphorylation, glycosylation, acetylation, and ubiquitination, enabling comprehensive interrogation of these modifications simultaneously. Compared with the traditional Clinical Proteomic Tumor Analysis Consortium workflow, MoSAIC doubles PTM coverage (four *versus* two PTMs) while maintaining the same instrument time (24 mass spectrometry runs), achieving increased identifications of PTM-modified peptides. By leveraging fractionation and tandem mass tag labeling, we achieved concurrent identification and quantification of PTM-specific peptides from the same sample, enhancing throughput and data consistency. This robust workflow addresses key limitations in multi-PTM proteomics, providing a cost-effective and efficient platform to advance biological and clinical research.

## Highlights

• Introduced acetylated/ubiquitinated peptide coenrichment to streamline workflow.
• Postenrichment TMT multiplexing enables DIA and preserves Gly-Gly antibody recognition.
• Pooled four PTM enrichments into one TMT mix, cutting redundant MS runs.
• Hybrid DDA/DIA with cross-validation reduces platform-specific bias.

## Graphical Abstract

**Figure:**
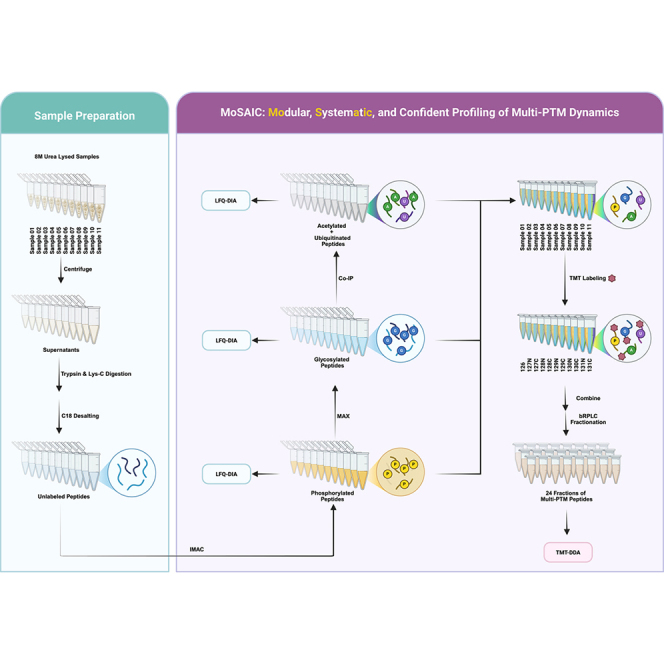

## In Brief

Post-translational modifications (PTMs) critically regulate protein function, yet simultaneous analysis of multiple PTMs remains technically challenging. We introduce MoSAIC, a modular and integrated workflow that combines targeted enrichment chemistries, multiplexed tandem mass tag labeling, and hybrid data-dependent acquisition/data-independent acquisition mass spectrometry for robust multi-PTM quantification. MoSAIC enables parallel profiling of phosphorylation, glycosylation, acetylation, and ubiquitination from the same biological sample, while reducing instrument time and improving reproducibility over existing approaches. Applied to breast cancer patient-derived xenografts, the workflow quantified >50,000 PTM-modified peptides and uncovered extensive PTM crosstalk. MoSAIC provides a scalable workflow for multidimensional PTM mapping with broad applications in translational research and biomarker discovery.

While the human genome is estimated to range from 20,000 to 25,000 coding genes (1), the human proteome bears several million proteins, with each gene producing multiple proteoforms (2). The proteome complexity is facilitated by different post-translational modifications (PTMs) and PTM crosstalk in each protein (3). PTMs serve as functional groups modulating protein functions as well as dysfunctions (4). Aberrant PTMs are often considered immediate indicators of such dysfunctions in response to internal or external stimuli (5). The understanding of which proteins are modified, at which sites they are modified, by which PTMs, and the corresponding physiological or pathological consequences is hence a subject worth investigating. Mass spectrometry (MS)–based proteomics has been a widely adopted approach to facilitate the investigation of complex subjects, with applications to PTM analysis (6, 7, 8). MS-based proteomics can detect and structurally define any covalent changes in a protein after translation.

With the explosion of new techniques and development of MS instrumentations in the past years, the number of PTM sites documented has accumulated to over 2 million (9). Phosphorylation, ubiquitination, acetylation, and glycosylation are the most extensively investigated PTMs (9). Phosphorylation is the most studied as well as one of the most characterized protein modifications (estimated to make up ∼30% of the proteome) (10), commonly occurring at serine, tyrosine, or threonine residues (11). Phosphorylation is indispensable in the regulation of various cellular processes, and the activation/deactivation of enzymes and receptors, which can also be implicated in pathological processes. Glycosylation is another prevalent (estimated to make up ∼50% of the proteome) yet the most complex PTM: a myriad of sugar moieties could be attached to/removed from specific amino acid residues, catalyzed by different enzymes (12). In addition, aberrant glycosylation is often observed in diseases such as cancer. Protein acetylation at lysine residues is a highly dynamic and specific PTM that frequently participates in protein–protein interactions, protein–DNA interactions, and protein catalytic activities (13). Ubiquitination is an important reversible PTM that serves to regulate protein functions or act as a protein degradation flag for proteasomes (14). Dysfunction in the ubiquitin pathway can contribute to disorders, such as cancer, metabolic syndromes, inflammatory disorders, type 2 diabetes, autoimmunity, and neurodegenerative diseases (15, 16, 17). Despite the recent technological advancements in MS-based proteomics, there are challenges that remain, such as low enrichment efficiencies of current PTM enrichment methodologies and a lack of robust analytical methods to detect multiple PTMs and decipher complex PTM data simultaneously.

An overarching problem with PTM proteomics studies is the transient nature of most PTMs, which often leads to low stoichiometry (<1–5%) (18). Enrichment of a PTM from the complex samples before MS analysis is crucial in leveraging the chemical properties of PTMs and facilitating targeted analysis (19). Current enrichment strategies in PTM studies are mostly utilizing the charge, hydrophilicity properties, or affinity-based properties specific to specific PTM levels (20). During enrichments, functional groups of the PTM are captured by an immobilized surface through reaction or affinity; unmodified fragments are usually washed away. However, different PTM studies often require different enrichment strategies with varying optimal conditions to ensure efficiency, which makes the multi-PTM study even more complex.

One of the quantitative PTM proteomic analyses involves tandem mass tag (TMT) labeling of global peptides followed by PTM-specific enrichment, primarily focusing on the phosphoproteome (21). However, TMT tagging limits downstream enrichment of certain additional PTMs, such as ubiquitinated peptides, as antibody recognition of Gly–Gly remnants is blocked by TMT tags. To enable multi-PTM analysis of over three PTMs, we developed an integrated multi-PTM analysis workflow to achieve integrated enrichments of phosphopeptides, glycopeptides, acetylated peptides, and ubiquitinated peptides as well as simultaneous data acquisition for quantitative measurement of these PTM peptides. This integrative workflow was applied to tumor tissues from breast cancer patient-derived xenograft (PDX) mouse models (22). Modification-specific peptides obtained from each step could be either labeled with TMT for isobaric-labeling quantitative MS or label-free data-independent acquisition (DIA) quantitative MS. TMT-labeled modification-specific peptides would then be pooled and fractionated to reduce the sample complexity. This innovative workflow stands out with its capability of analyzing four PTMs simultaneously with a simplified enrichment procedure and less instrument time. The step-by-step nature of this workflow also allows us to perform DIA analysis on top of data-dependent acquisition (DDA) analysis with the benefit of enhanced identification confidence and verification of PTMs by DIA analysis. This pipeline has the potential of including PTMs beyond phosphorylation, glycosylation, acetylation, and ubiquitination. Allowing us to robustly identify and quantify modification-specific peptides that are of statistical significance, also proteins with more than one modification.

## Experimental Procedures

### Experimental Model and Subject Details

PDX tumors from established basal and luminal breast cancer models (23) were used in this study. The xenograft tumors were grown subcutaneously in 8-week-old NOD.Cg-*Prkdc*^*scid*^ *Il2rg*^*tm1Wjl*^/SzJ mice (Jackson Laboratories, strain code 005557). These PDX models were obtained from the National Cancer Institute Patient-Derived Models Repository and are available upon request through the Human and Mouse-Linked Evaluation of Tumors Core (http://digitalcommons.wustl.edu/hamlet/). All animal works were performed by the Patient-Derived Models Repository under approved institutional protocols, including the National Cancer Institute Tissue Procurement Protocol (ClinicalTrials.gov identifier: NCT00900198) and Central Institutional Review Board Protocol 9846. No animal procedures were conducted directly by the authors of this study. For processing, tumor pieces from each subtype were cryopulverized, and the tissue powder obtained was stored at −80 °C.

### Tryptic Digestion

Breast cancer PDX mouse models, basal-like subtypes, and luminal subtypes were lysed using 8 M urea lysis buffer (8 M urea, 75 mM NaCl, 50 mM Tris–HCl, pH 8), and protein concentration was determined using Bicinchoninic Acid Assay kit (23225, Pierce Biotechnology). Enzymatic tryptic digestion was performed as described previously (24). Digested samples were desalted by C18 (WAT036820, Sep-Pak tC18 Cartridge, Waters) and dried using Speed-Vac.

### Immobilized Metal Affinity Chromatography Enrichment for Phosphopeptides

Dried peptides were suspended in 3% (v/v) acetonitrile (ACN), 0.1% (v/v) TFA, and the peptide concentration was measured using Nanodrop (Thermo Scientific). Aliquots of ∼300 μg peptides were then reconstituted into 600 μl of 80% (v/v) ACN, 0.1% (v/v) TFA (peptide concentration controlled at ∼0.5 μg/μl) for the following immobilized metal affinity chromatography (IMAC) enrichment. From this, phosphopeptide enrichment *via* IMAC typically yielded three injections for LC–MS/MS.

IMAC procedure was performed using Fe^3+^–nitrilotriacetic acid (NTA) agarose beads that were freshly prepared using Ni^2+^–NTA agarose beads (QIAGEN, catalog no.: 30210) as previously described (25). Samples constituted in 80% (v/v) ACN and 0.1% (v/v) TFA were incubated with 100 μl of 5% (v/v) Fe^3+^–NTA agarose beads to conjugate for 30 min at room temperature. After conjugation, the supernatant containing unbound peptides was collected by centrifugation. The beads with conjugated peptides were carefully transferred onto a C18 Stage Tip and washed three times with 200 μl 80% (v/v) ACN and 0.1% (v/v) TFA. The washes were combined with the supernatant for the subsequent enrichment process. The peptides conjugated to beads were eluted with 100 μl potassium phosphate buffer (500 mM KH_2_PO_4_, pH 7) for three times and 50% (v/v) ACN and 0.1% (v/v) formic acid (FA). Eluted phosphopeptides and unbound peptides from IMAC were dried and stored at −80 °C for LC–MS/MS analysis.

### MAX Enrichment for Intact Glycopeptides

For the enrichment of intact glycopeptides (IGPs), MAX cartridges (Oasis MAX cartridge 30 mg, Waters) were used as previously described (26). Unbound peptides from the IMAC enrichment process were reconstituted with 1 ml of 95% (v/v) ACN and 1% (v/v) TFA. The MAX cartridge was conditioned with three times 1 ml of ACN, three times 1 ml of 100 mM TAAB buffer, four times 1 ml of 95% (v/v) ACN, 1% (v/v) TFA, respectively, and the peptide mixture was subsequently loaded onto the cartridge two times. Flow-through was collected. The MAX cartridge was then washed four times with 1 ml of 95% (v/v) ACN and 1% (v/v) TFA, and the washes were combined with the flow-through for the subsequent enrichment process. The peptides conjugated onto MAX were eluted using 50% (v/v) ACN and 0.1% (v/v) TFA. Eluted IGPs and unbound peptides from MAX were dried and stored at −80 °C for LC–MS/MS analysis. The eluted IGPs were reconstituted and split into three equal injections for LC–MS/MS analysis; unbound peptides were subject to subsequent enrichments.

### Immunoprecipitation for Ubiquitinated and Acetylated Peptide Enrichment

The dried peptides unbound during the MAX enrichment were reconstituted in 600 μl of the immunoaffinity purification (IAP) buffer (50 mM Mops/NaOH [pH 7.2], 10 mM Na_2_HPO_4_, and 50 mM NaCl). The pH of the peptide solution was then checked with pH indicator paper (Whatman). The antibody beads from PTMScan Acetyl-Lysine Motif [Ac-K] Kit (Cell Signaling, #13416S) or PTMScan Ubiquitin Remnant Motif (K-ε-GG) Kit (Cell Signaling, #5562S) were freshly prepared immediately before the incubation. Briefly, the antibody beads were centrifuged at 2000*g* for 30 s, and all buffers from the beads were removed; the antibody beads were then washed with 1 ml of IAP buffer four times and finally resuspended in 40 μl of IAP buffer. For each sample, a quarter of the antibody in each tube was transferred to the peptide solution and incubated on a rotator overnight at 4°C. After removing the supernatant, the reacted beads were resuspended using 600 μl of ice-cold IAP buffer and transferred onto a column system (Thermo Fisher, #69725). The beads were washed with 1 ml ice-cold IAP four times. For the elution of peptides from the antibody beads, 50 μl of 0.15% TFA was used to incubate the antibody beads at room temperature for 10 min with occasional flicks on the tubes. The eluted peptides were collected through centrifugation. Repeat the elution process one more time (two cycles in total). The eluted peptides were then transferred onto a conditioned C18 Stage Tip for desalting. Peptides were eventually eluted with 100 μl 50% (v/v) ACN and 0.1% (v/v) TFA twice and dried using Speed-Vac and stored at −80 °C for LC–MS/MS analysis. From 300 μg of input peptides, each acetyl- and ubiquitin-IP typically yielded sufficient material for three LC–MS/MS injections.

### TMT Labeling and Basic Reverse-Phase Fractionation

Six basal-like and five luminal peptide–enriched samples were labeled with TMT 11-plex reagents (Thermo Scientific) under previously optimized conditions, achieving near-complete labeling (>95% of peptides observed as fully labeled) (21). Basal-like samples were labeled with 126, 127N, 127C, 128N, 128C, and 129N, and luminal samples were labeled with 129C, 130N, 130C, 131N, and 131C of TMT channels. TMT-11plex-labeled peptides were separated on a reversed-phase Zorbax Extend-C-18 column (4.6 × 100 mm, 1.8 μm particles, Agilent Technology) using an Agilent 1260 Infinity HPLC System. HPLC gradient condition was set as follows with solvent A (10 mM ammonium formate, pH 10) and solvent B (10 mM ammonium formate in 90% [v/v] ACN, pH 10): 2% (v/v) B for 10 min, from 2% to 8% B for 5 min, from 8% to 35% B for 85 min, from 35% to 95% B for 5 min, and 95% B for 25 min. Eluents were collected on the 96-well plate with 0.2 ml/min and concatenated into the desired number of fractions and dried using Speed-Vac.

### LC–MS/MS Analysis

#### DDA Analysis

The analytical column was manufactured in-house using ReproSil-Pur 120 C18-AQ 1.9 μm stationary phase (Dr Maisch GmbH) and slurry packed into a 28-cm length of 360 μm o.d. x 75 μm i.d. fused silica PicoFrit capillary tubing (New Objective). The analytical column was heated to 50 °C using a column heater (Phoenix-ST). The analytical column was equilibrated to 98% mobile phase A (3% [v/v] ACN, 0.1% [v/v] FA) and 2% mobile phase B (MP B, 90% [v/v] ACN and 0.1% [v/v] FA) and maintained at a constant column flow of 200 nl/min. The sample was injected into a 12 μl loop placed in-line with the analytical column, which initiated the gradient profile (minimum: %MP B): 0:2, 1:6, 85:30, 94:60, 95:90, 100:90, 101:50, and 110:50. The column was allowed to equilibrate at start conditions for 30 min between analytical runs.

MS analysis was performed using an Orbitrap Fusion Lumos mass spectrometer (Thermo Fisher Scientific). The phosphoproteome, acetylome, and ubiquitinome samples were analyzed under identical conditions. Electrospray voltage (1.8 kV) was applied at a carbon composite union (Valco Instruments) coupling a 360 μm o.d. x 20 μm i.d. fused silica extension from the LC gradient pump to the analytical column, and the ion transfer tube was set at 305 °C. Following a 25-min delay from the time of sample injection, Orbitrap precursor spectra (automatic gain control [AGC] 4 x 10^5^) were collected from 350 to 1800 *m/z* for 110 min at a resolution of 60 K along with data-dependent Orbitrap high-energy collision dissociation (HCD) MS/MS spectra (centroid) at a resolution of 50 K (AGC 2 x 10^5^) and maximum injection time of 105 ms for a total duty cycle of 2 s. Masses selected for MS/MS were isolated (quadrupole) at a width of 0.7 *m/z* and fragmented using a collision energy of 37%. Peptide mode was selected for monoisotopic precursor scan, and charge state screening was enabled to reject unassigned 1+, 7+, 8+, and >8+ ions with a dynamic exclusion time of 45 s to discriminate against previously analyzed ions between ±10 ppm. The glycoproteome and MoSAIC samples were analyzed under similar conditions except that the collision energy is set to 38%.

#### Spectral Library Generation for DIA–MS Analysis of IGPs

For spectral library generation, two pooled samples were created: one from five individual basal samples (each 1 μg) and another from five individual luminal samples (each 1 μg). Each pooled sample was spiked with indexed retention time (iRT) peptides (Biognosys) before electrospray ionization–LC–MS/MS analysis, with each sample being analyzed three times.

The LC gradient used was as follows (time in minutes: % mobile phase B): 0:2, 3:7, 93:25, 121:30, 125:60, 126:90, 130:90, 131:50, and 140:50. The analysis was performed on an Orbitrap Fusion Lumos mass spectrometer (Thermo Scientific) with the following parameters: MS1: resolution—60 K, mass range—350 to 2000 *m/z*, RF lens—30%, AGC target 4.0e5, maximum injection time—50 ms, charge state include—2 to 6, dynamic exclusion—45 s, top 20 ions selected for MS2; MS2: resolution—15 K, HCD activation energy—34, isolation width (*m/z*)—0.7, AGC target—2.0e5, and maximum injection time—105 ms. The obtained raw files were searched against the UniProt human database (20,418 entries, downloaded in February 2019) and a human glycosylation database (36,218 glycosites and 253 glycan compositions) using GPQuest. A glycosylation library, consisting of 764 precursors (Supplemental Table S3), was subsequently generated from the search results and imported into Spectronaut (version 13.9.19), following the procedure described in our previous study (27).

#### DIA Analysis

Unlabeled, modification-specific peptides obtained from individual tissue samples (basal or luminal PDX) were spiked with iRT peptides (Biognosys) and subjected to DIA analysis. Peptides were separated on an Easy nLC 1200 UHPLC system (Thermo Scientific) on an in-house packed 28-cm length of 360 μm o.d. x 75 μm i.d. fused silica PicoFrit capillary tubing (New Objective). The column was heated to 50°C using a column heater (Phoenix-ST). The flow rate was 0.200 ml/min with 3% (v/v) ACN, 0.1% (v/v) FA (mobile phase A) and 90% (v/v) ACN, 0.1% (v/v) FA (MP B). The phosphopeptides were separated using the following LC gradient: (minimum: %MP B): 0:2, 1.5:7, 44:25, 58:30, 60:60, 61:90, 65.5:50, and 70:50. The glycopeptides, acetylated peptides, and ubiquitinated peptides were separated using the following LC gradient: (minimum: %MP B): 0:2, 3:7, 93:25, 121:30, 125:60, 126:90, 130:90, 131:50, and 140:50. MS analysis was performed using an Orbitrap Fusion Lumos mass spectrometer (Thermo Fisher Scientific). For phosphoproteome, acetylome, and ubiquitinome, the DIA segments consisted of one MS1 scan (350–1650 *m/z* range, 120 K resolution) followed by 30 MS2 scans (variable *m/z* range, 30 K resolution) as described previously (28). For the glycoproteome, DIA segments consisted of one MS1 scan (450–1650 *m/z* range, 120 K resolution) followed by 50 MS2 scans (variable *m/z* range, 15 K resolution) as described previously (27).

#### Data Processing

All DDA .raw files from label-free and TMT-labeled data were processed through MS-PyCloud (version 2.10.1) (29), with the following parameters: fixed modifications of carbamidomethyl at cysteine and TMT at lysine and peptide N terminus, dynamic modification of oxidation at methionine, precursor mass tolerance of 20 ppm, missed cleavages ≤2, instrument ID of “High-res LTQ” (default fragment ion mass tolerance is set to ±0.02 Da), and fragmentation method of HCD. A false discovery rate (FDR) of 1% was applied at the peptide-spectrum match (PSM) level, with a minimum requirement of one PSM per peptide and one peptide per protein. Protein database searches were performed using a combined human and mouse SwissProt database (37,404 entries, downloaded in December 2019).

Database searches for phosphopeptides, glycopeptides, acetylated peptides, and ubiquitinated peptides were performed independently, restricting dynamic modifications to the targeted PTM and common modifications. Potential overlaps across searches were also evaluated and excluded from downstream analyses. This targeted search strategy reduces the search space and minimizes false positives from unrelated modifications. For each PTM, a target-decoy approach was used to estimate and control the PSM-level FDR at 1% prior to peptide and protein inference. Only PSMs meeting the FDR threshold and, where applicable, passing PTM site localization confidence filtering were included in downstream quantification. PTM-specific feature abundances were determined by summing the abundances of PSMs associated with the same PTM-specific feature. Each TMT channel was normalized using the column median, and the data matrix was log2-transformed prior to analysis.

The DIA .raw files of phosphoproteome, acetylome, and ubiquitinome were searched using the directDIA in Spectronaut (version 16.2; Biognosys). For phosphoproteome analysis, carbamidomethylation of cysteine was set as a fixed modification, whereas oxidation of methionine, acetylation at the protein N terminus, and phosphorylation at serine, threonine, and tyrosine were specified as variable modifications; up to two missed cleavages were allowed. For acetylome and ubiquitinome analyses, carbamidomethylation of cysteine and the respective PTM (lysine acetylation for acetylome, K-ε-GG ubiquitin remnant for ubiquitinome) were set as fixed modifications, whereas oxidation of methionine and protein N-terminal acetylation were set as variable modifications; up to two missed cleavages were allowed. The DIA .raw files of the IGPs were analyzed using Spectronaut (version 16.2; Biognosys) against a spectral library (764 precursors) generated from GPQuest searches of DDA. raw files (27). Mass tolerance of MS and MS/MS was set as dynamic with a correction factor of 1. Source-specific iRT calibration was enabled with local (nonlinear) RT regression. All multichannel interferences were excluded, and the decoy method was set as ‘‘mutated.” The precursors were filtered by a *Q*-value cutoff of 0.01 (which corresponds to an FDR of 1%). The quantity of a modified peptide was decided by summing the quantity of its precursors, whereas the quantity of a precursor was calculated by summing the area of its fragment ions at the MS2 level. The reported quantification result was filtered as previously described (27). In brief, the filtering criteria consisted of the following: the full width at half maximum of the extracted ion chromatogram of the fragment ions <1 min, the shape quality score for the extracted ion chromatogram of the precursor transition groups >0.6, signal-to-noise ratio of the fragment ions >3, and cosine similarity between theoretical and measured isotopic patterns of precursors >0.9. The missing values were imputed using the KNN algorithm, where only peptides with a missing rate less than 50% across all samples were imputed for quantifications.

#### Experimental Design and Statistical Rationale

This study analyzed proteomic differences between basal-like and luminal breast cancer subtypes using PDX mouse models. For each subtype, a PDX tissue sample was used to generate representative lysates. Multiple aliquots from each lysate were independently processed through the multi-PTM workflow, resulting in six technical replicates for the basal subtype and five technical replicates for the luminal subtype. This design enables the evaluation of analytical reproducibility and workflow robustness as well as the quantitative differences between the two subtypes. As these replicates were derived from the same pooled lysate, they represent technical replicates, not biological replicates.

Quantitative intensities of PTM-specific sites were obtained by summing the intensities of PSMs assigned to the same PTM site, followed by log2 transformation and median normalization across TMT reporter ion channels. For reproducibility assessment, the CV was calculated at the PTM-site level as the standard deviation divided by the mean of normalized intensities across all 11 TMT channels (six basal + five luminal). The distribution of overall site-level CVs for the phosphoproteome, glycoproteome, acetylome, and ubiquitinome was visualized using stacked violin plots in the result section.

#### Differential Abundance Analysis

Phosphoproteomic, glycoproteomic, acetylomic, and ubiquitinomic datasets were analyzed independently to isolate subtype-specific differences. A two-sample *t* test was used to perform pairwise differential analysis between basal and luminal subtypes. At least three samples in both groups were required to have nonmissing values, and the *p* value was adjusted using the Benjamini–Hochberg procedure, and features were considered significant with an adjusted *p* value <0.05. PTM-specific peptides with at least a twofold increase/decrease were deemed to be subtype-associated differences.

#### Cross-Platform Comparison

To examine directional agreement of PTM-specific features across different proteomic platforms, we performed a cross-platform comparison between TMT–DDA and label-free quantification (LFQ)–DIA datasets. Following differential abundance analysis, PTM-specific features that were mutually identified in both datasets were selected for evaluation. Significantly regulated PTM-specific features (n = 550) were mapped to 384 unique proteins and subjected to STRING network analysis and Gene Ontology (GO) enrichment to identify enriched biological processes, molecular functions, and cellular components. In addition, the log2 fold changes (FCs) derived from TMT–DDA and LFQ–DIA were visualized using two complementary approaches: (i) nine-quadrant analysis of mutually identified and quantified PTM-specific peptides, where all shared peptides are shown without *p* value filtering to allow an unbiased comparison of directional changes between methods and (ii) scatter plots of log2 FCs with marginal density plots, where Spearman's correlation coefficients (ρ) were calculated separately for phosphoproteomic, glycoproteomic, acetylomic, and ubiquitinomic datasets to evaluate cross-platform consistency .

## Results

### Development of an Integrated Multi-PTM Workflow

**Fig. 1:**
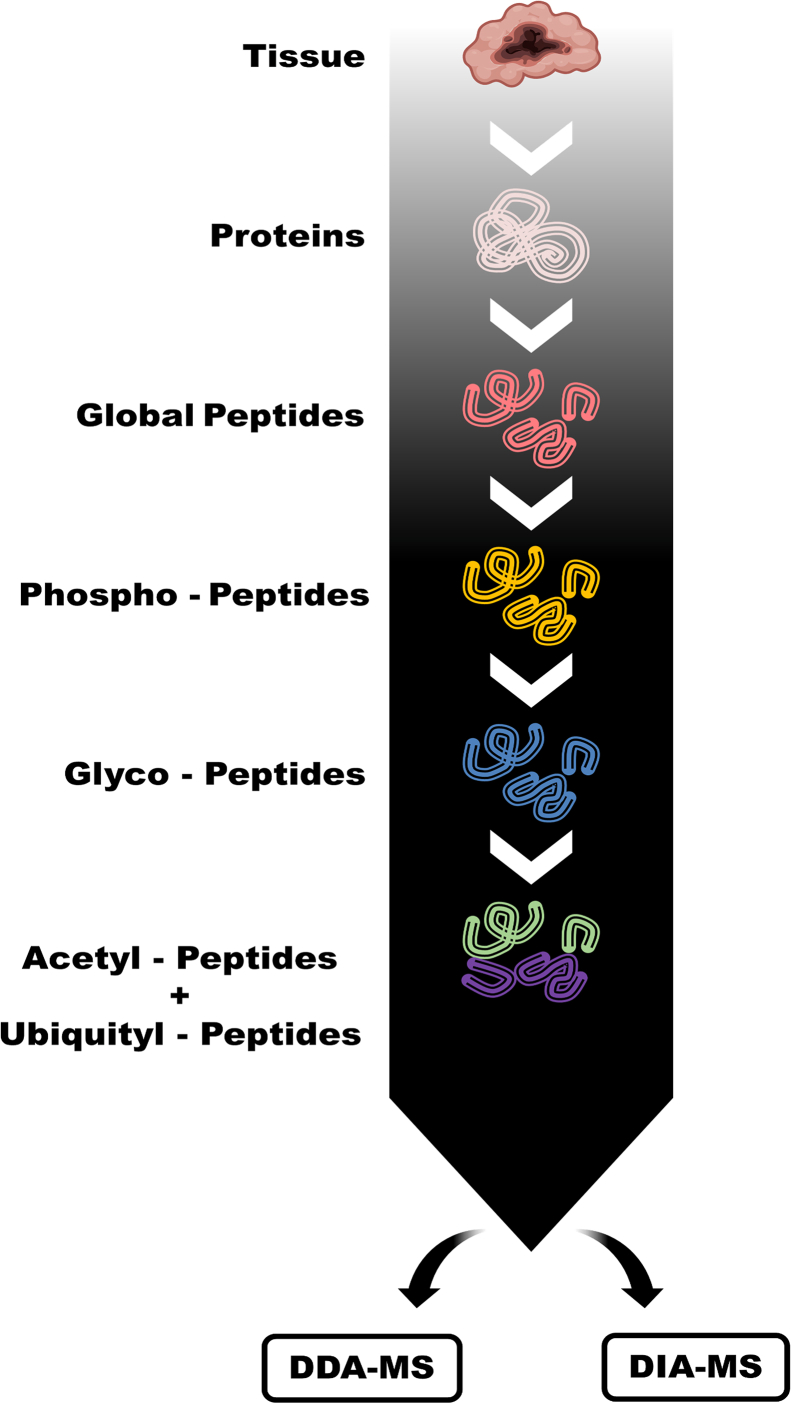
**MoSAIC multi-PTM workflow**. Protease-digested peptides derived from biological or clinical samples are subject to serial enrichments of phosphopeptides, glycopeptides, and acetylated peptides/ubiquitinated peptides. Enriched PTM peptides from each sample are labeled with a tandem mass tag. After labeling, samples were fractionated and analyzed by LC–MS/MS using data-dependent acquisition. In addition, data-independent acquisition analysis could be directly adopted for samples obtained from each enrichment step. Key innovations: (1) coenrichment of acetylated/ubiquitinated peptides; (2) tandem mass tag labeling postenrichment; and (3) hybrid data-dependent acquisition/ data-independent acquisition. PTM, post-translational modification.

To address the unmet need of simultaneous analysis of multiple PTMs, we developed a novel workflow incorporating coenrichment strategies, multiplexing, fractionation, hybrid data acquisition, and unified data analysis for the study of multiple PTMs from the same sample (Fig. 1). Our workflow starts with the lysis of biological or clinical samples into proteins. Proteins were then digested using proteases into peptides. Phosphopeptides were enriched from the global peptides, followed by the enrichment of glycopeptides from the flow-through from phosphopeptide enrichment (30). The flow-through from the serial enrichments of phosphopeptides and glycopeptides was used for the simultaneous immunoprecipitation of acetylated peptides and ubiquitinated peptides. Prior to large-scale application, the feasibility of simultaneous enrichment of acetylated and ubiquitinated peptides was validated to ensure compatibility (Supplemental Fig. S1).

After serial enrichments, 50% of the PTM-specific peptides were labeled with respective TMT tags, combined, and subject to offline basic reversed-phase (bRP) fractionation prior to DDA analysis. At high pH, bRP fractionation is highly orthogonal with the LC–MS/MS analysis using low-pH reversed-phase chromatography in-line with the mass spectrometer. With the additional dimension introduced by offline bRP fractionation, the complexity of the sample was reduced significantly by separating peptides into different classes based on their varied biophysical properties. The simultaneous DDA analysis of a multi-PTM sample was thus made more accessible. The remaining 50% of the enriched PTM peptides were used for DIA analysis.

### Identification Depth of the Workflow for Different PTMs

**Fig. 2:**
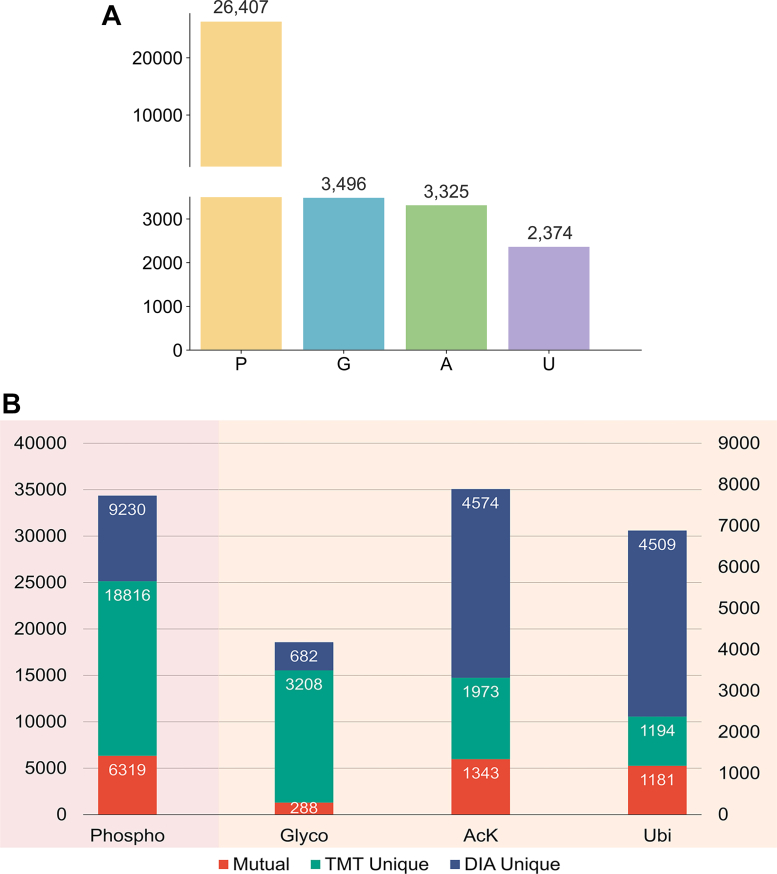
**Robust analysis of multi–post-translational modifications**. *A*, bar graph of the post-translational modification–specific identifications from a tandem mass tag–based data-dependent acquisition dataset. *B*, stacked bar graph of the post-translational modification–specific identifications from tandem mass tag–based data-dependent acquisition dataset and data-independent acquisition dataset.

To evaluate the performance of our multi-PTM workflow MoSAIC, we applied it to tumor tissues from breast cancer PDX mouse models (22). Six basal-like and five luminal PDX tumor tissues were lysed, enzymatically digested, and subjected to serial PTM enrichment (phosphorylation → glycosylation → acetylation/ubiquitination). Enriched phosphopeptides, glycopeptides, acetylated peptides, and ubiquitinated peptides from each sample were pooled and labeled by one of the TMT-11 channels. The 11 TMT-labeled samples were further combined and fractionated *via* offline bRPLC into 24 fractions to reduce sample complexity prior to analysis by subsequent TMT–DDA. From the TMT–DDA dataset, we identified 26,407 phosphopeptides, 3496 glycopeptides, 3325 acetylated peptides, and 2374 ubiquitinated peptides. The robust PTM-specific peptide identifications across both basal and luminal subtypes underscore the workflow’s capability in resolving complex proteoform landscapes (Fig. 2*A*).

To complement the TMT–DDA platform and mitigate method-specific biases, we employed LFQ–DIA as an orthogonal MS approach. The LFQ–DIA dataset yielded 15,661 phosphopeptides, 682 glycopeptides, 5930 acetylated peptides, and 5695 ubiquitinated peptides. By integrating results from both TMT–DDA and LFQ–DIA, we achieved a total of 35,352 phosphopeptides, 3890 glycopeptides, 7919 acetylated peptides, and 6888 ubiquitinated peptides (Fig. 2*B*). This hybrid approach enhanced coverage while minimizing redundancy, underscoring the complementary strength of TMT–DDA and LFQ–DIA in multi-PTM profiling.

### Analytical Performance of Our Multi-PTM Workflow Over Alternative Pipelines

**Fig. 3:**
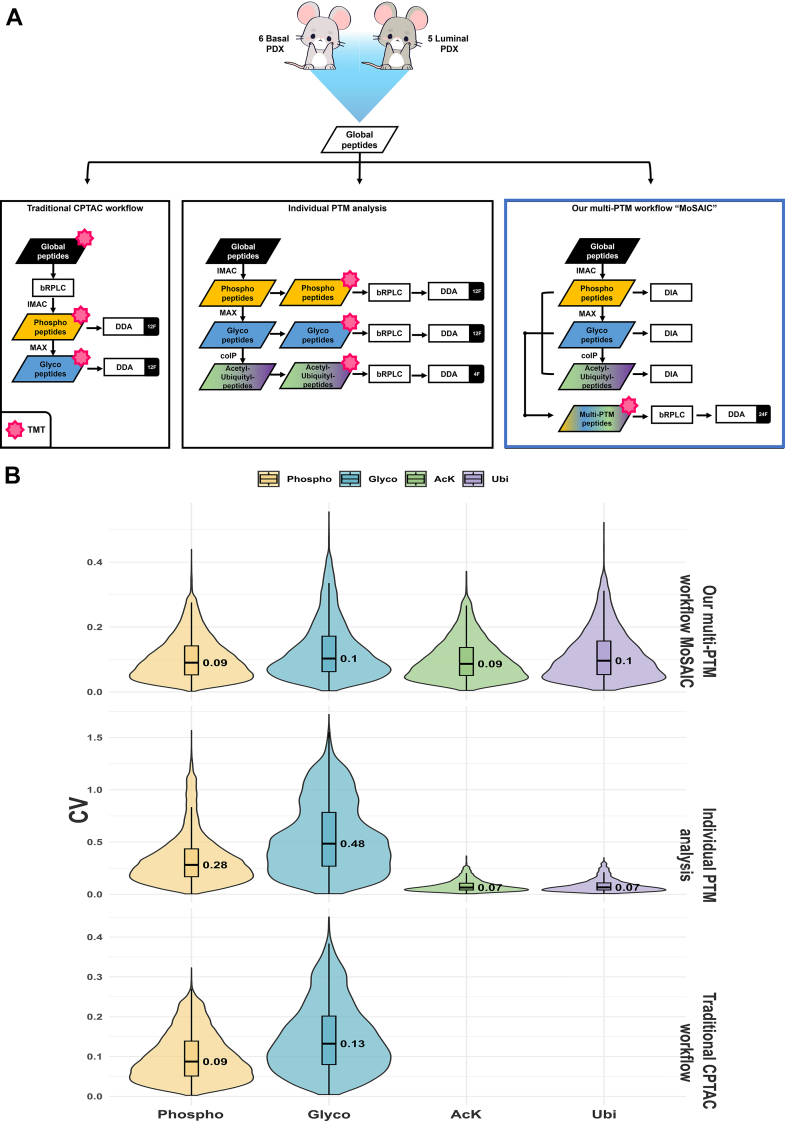
**Evaluation of multi-PTM pipelines**. *A*, comparison of multi-PTM pipelines. *Left* (traditional Clinical Proteomic Tumor Analysis Consortium workflow): “Global” concatenation, peptides derived from basal-like and luminal PDX samples were labeled with tandem mass tags (TMTs) before fractionation, then each of the fractions was subject to serial enrichment of phosphopeptides and glycopeptides. *Middle* (individual PTM analysis): peptides derived from basal-like and luminal PDX samples were subject to serial enrichments of phosphopeptides, glycopeptides, and acetyl-peptides/ubiquityl-peptides. Samples obtained from each PTM enrichment step were labeled by TMT and fractionated. *Right* (multi-PTM workflow “MoSAIC”): peptides derived from basal-like and luminal PDX samples were subject to serial enrichments of phosphopeptides, glycopeptides, and acetyl-peptides/ubiquityl-peptides. Different PTM-enriched peptides from each sample were mixed and labeled with TMT. After labeling, all TMT channels were concatenated and fractionated for LC–MS/MS analysis using data-dependent acquisition. In addition, data-independent acquisition analysis could be directly adopted for samples obtained from each enrichment step. *B*, stacked violin plot of CV for phosphoproteome, glycoproteome, acetylome, and across different pipelines. PDX, patient-derived xenograft; PTM, post-translational modification.

To assess the analytical performances of our newly developed workflow MoSAIC, we systematically evaluated our workflow against two alternative multi-PTM pipelines (Fig. 3*A*): (1) the traditional Clinical Proteomic Tumor Analysis Consortium workflow (*left*) (21), where global peptides were first TMT labeled, pooled, fractionated, and subjected to serial enrichments of phosphopeptides (12 fractions) and glycopeptides (12 fractions). While this approach profiles two PTMs, it requires 24 MS runs; (2) individual PTM analysis (*middle*), involving serial enrichments for phosphopeptides, glycopeptides, acetylated and ubiquitinated peptides from individual samples. Enriched PTM-specific peptides from individual samples were labeled with one of the TMT-11 channels. After TMT labeling, enriched PTM-specific peptides from 11 PDX samples were combined and fractionated by PTM type (12, 12, and 4 fractions for phosphopeptides, glycopeptides, and acetylated/ubiquitinated peptides). This method profiles four PTMs but requires 28 MS runs; (3) our multi-PTM workflow MoSAIC (*right*), which integrates serial enrichment of phosphorylation, glycosylation, acetylation, and ubiquitination from the same sample, followed by pooling of all PTM-specific peptides by sample origin, labeling each pooled sample with a unique TMT-11 tag, and combining labeled samples into a single mixture for downstream fractionation (24 fractions) of all PTM-specific peptides. This streamlined process profiles four PTMs with only 24 MS runs, matching the traditional Clinical Proteomic Tumor Analysis Consortium workflow’s runtime while doubling PTM coverage (21).

The comparison of the three workflows revealed that our selected pipeline yielded superior identifications for glycopeptides (3496 *versus* 2759/2220), acetylated peptides (3325 *versus* not applicable/2099), and ubiquitinated peptides (2374 *versus* not applicable/1121), whereas maintaining comparable identifications for phosphopeptides (26,407 *versus* 27,023/23,270, Table 1). Moreover, our chosen workflow exhibited better reproducibility, showing lower CVs across the phosphoproteome (9% *versus* 9%/28%), glycoproteome (10% *versus* 13%/48%), acetylome (9% *versus* not applicable/7%), and ubiquitinome (10% *versus* not applicable/7%) (Fig. 3*B*), underscoring its robustness and reproducibility. Collectively, these results highlight the efficacy of our multi-PTM workflow in achieving comprehensive and high-confidence multi-PTM profiling.

**Table 1:** Numbers of identified phosphopeptides, glycopeptides, acetylated peptides, and ubiquitinated peptides across all three TMT–DDA pipelines

Workflow	Phosphopeptides	N-linked glycopeptides	Acetylated peptides	Ubiquitinated peptides
Traditional CPTAC workflow	27,023	2759	N/A	N/A
Individual PTM analysis	23,270	2220	2099	1121
Our multi-PTM workflow “MoSAIC”	26,407	3496	3325	2374

Clinical Proteomic Tumor Analysis Consortium (CPTAC) workflow does not support coenrichment of acetylated/ubiquitinated peptides.

N/A, not applicable.

Our proposed pipeline (*right*, multiPTM workflow “MoSAIC”) for profiling phosphorylation, glycosylation, acetylation, and ubiquitination demonstrated several key advantages over the two alternative TMT–DDA workflows. Notably, it uniquely enables simultaneous and comprehensive profiling of all four PTMs without compromising peptide identifications or quantifications. Furthermore, this workflow exhibits the flexibility to accommodate additional PTMs and integrate other acquisition schemes, such as DIA (Fig. 2*B*).

### High-Confidence Profiling of PTM Dynamics in PDX Models Using Both TMT–DDA and LFQ–DIA Quantitative Results

**Fig. 4:**
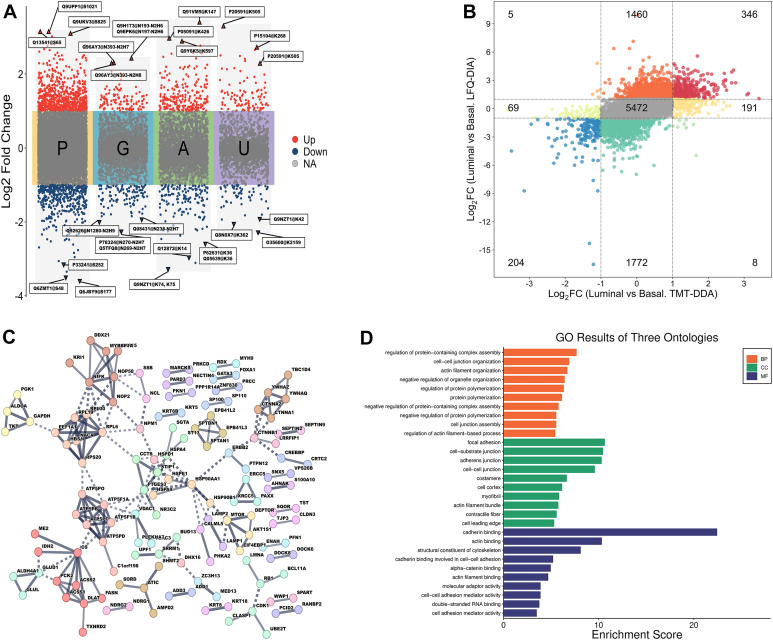
**Quantitative analysis of multi-PTMs using DDA and DIA**. *A*, differential analyses of phosphoproteome (P), glycoproteome (G), acetylome (A), and ubiquitinome (U) generated from our multi-PTM pipeline using the TMT–DDA analysis: upregulated data points were marked in *red*, and downregulated data points were marked in *blue*. The shaded background in *gray color* indicates the ranges of log2 fold change without statistical significance. *B*, nine-quadrant analysis of the PTM-specific peptides mutually identified and quantified in both TMT–DDA and LFQ–DIA datasets. All shared peptides are shown without *p* value filtering to allow an unbiased comparison of directional changes between methods. *C*, STRING analysis was performed on 384 proteins corresponding to 550 significant PTM-specific peptides. Nodes represent proteins, and edges denote associations with a confidence score ≥0.7 (sources: experimental, database, coexpression, neighborhood, gene fusion, and co-occurrence). The network was clustered using the MCL algorithm (inflation = 3), and interaction modules were color coded. Visualization and annotations were performed in STRING (https://string-db.org; accessed March 2024). *D*, Gene Ontology (GO) analysis of three ontologies based on the 550 significantly regulated PTM-specific peptides consistently identified from TMT–DDA and LFQ–DIA. DDA, data-dependent acquisition; DIA, data-independent acquisition; LFQ, label-free quantification; MCL, Markov Clustering; PTM, post-translational modification; TMT, tandem mass tag.

**Supplemental Figure S2:**
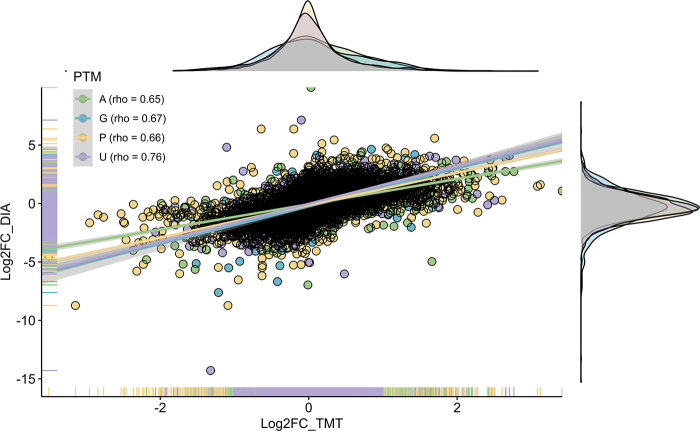
Scatter-density plot of the log2 fold change of TMT-DDA results and DIA results for phosphoproteome (P, Spearman Rho = 0.66), glycoproteome (G, Spearman Rho = 0.67), acetylome (A, Spearman Rho = 0.65) and ubiquitome (U, Spearman Rho = 0.76).

Single-platform proteomic workflows are inherently prone to method-specific biases and stochastic limitations. To overcome these limitations and achieve high-confidence PTM profiling, we performed cross-platform validation to ensure PTM identifications and quantifications, significantly enhancing confidence in biologically relevant findings. We first performed differential analysis of luminal *versus* basal-like PDX tumors using TMT–DDA data acquired on an Orbitrap Fusion Lumos mass spectrometer. A two-sample *t* test identified 1083 phosphopeptides, 215 glycopeptides, 331 acetylated peptides, and 150 ubiquitinated peptides with significant regulation (|log2FC| >1, Benjamini–Hochberg adjusted *p* < 0.05; Fig. 4*A*, Supplemental Table S1). However, to ensure these findings were not biased and identify PTM-specific peptides that were significantly regulated with high confidence, we complemented the TMT–DDA analysis with label-free DIA-MS, an orthogonal quantitative approach. The remaining 50% of individually enriched PTM samples without TMT labeling were analyzed by DIA–MS on the same Orbitrap Fusion Lumos mass spectrometer. A nine-quadrant analysis of log2 FCs was conducted between TMT–DDA and LFQ–DIA–results (Fig. 4*B*). This comparative analysis yielded 550 significantly regulated PTM-specific peptides (TMT–DDA |log2 FC| >1 and LFQ–DIA |log2 FC| >1): 311 phosphopeptides, 27 glycopeptides, 124 acetylated peptides, and 88 ubiquitinated peptides. Spearman's correlation analysis of log2 FCs of PTM-specific peptides confirmed quantitative agreement (ρ > 0.65) between platforms (Supplemental Fig. S2).

To further explore the biological context of these findings, STRING analysis was performed on 384 proteins corresponding to the 550 identified PTM-specific peptides (Fig. 4*C*). This analysis revealed an intricate protein–protein interaction network, characterized by associations with a confidence score of ≥0.7, derived from multiple evidence sources. The network was partitioned into distinct modules using the Markov Clustering algorithm (inflation = 3) (31), with key clusters color coded for visualization. Among these, the pyruvate metabolism cluster (colored in red) exhibited the highest coverage, covering proteins integral to central energy production and biosynthetic pathways. This finding underscores the critical role of pyruvate metabolism in regulating PTM-associated cellular processes and highlights its potential as a metabolic hub for integrating PTMs. Additional clusters were linked to ATP synthesis, ribosomal assembly, peptide chain elongation, and host–pathogen interactions, reflecting the diverse functional landscape influenced by PTM-specific peptides.

GO enrichment analysis of these peptides revealed key biological processes, cellular components, and molecular functions impacted by multi-PTM regulations (Fig. 4*D*). These GO terms have been implicated in cancer progression and metastasis. For instance, disruption of cell–cell junction organization plays a pivotal role in tumor growth and metastasis, emphasizing its contribution to intercellular adhesion and signaling (32). The regulation of actin filament organization is crucial for maintaining cellular architecture and promoting metastatic behavior in breast cancer cells, particularly in the context of cytoskeletal dynamics (33).

**Supplemental Figure S3:**
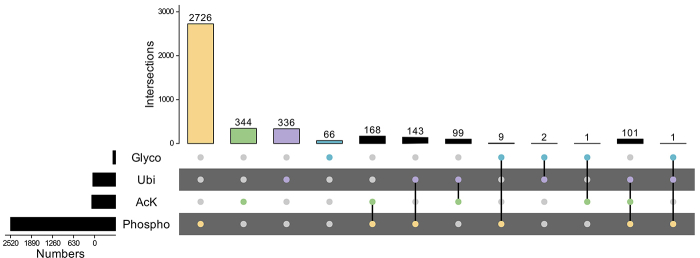
UpSet plot of proteins consistently identified in TMT and DIA data.

We next investigated the co-occurrence of multiple PTMs on individual proteins to uncover potential regulatory synergies. Analysis of PTM-specific peptides mutually identified in both DIA and TMT datasets revealed extensive crosstalk between different PTMs, with hundreds of proteins exhibiting more than one modification (Supplemental Fig. S3). Notably, 168 proteins were modified by both phosphorylation and acetylation, and 143 proteins showed modifications by phosphorylation and ubiquitination. Phosphorylation and glycosylation were identified on nine proteins, whereas 99 proteins carried both acetylation and ubiquitination. A total of 101 proteins are modified by a combination of phosphorylation, acetylation, and ubiquitination. Intriguingly, the cross-platform analysis discovered several uncharacterized PTM combinations on clinically relevant breast cancer proteins that are absent in UniProt. Specifically, SCGB2A2 (mammaglobin-A)—a luminal biomarker used in the FDA-cleared GeneSearch Breast Lymph Node assay for metastasis surveillance (US Food and Drug Administration, 2007) (34), was found to be glycosylated (N53) and ubiquitinated (K39). These modifications may modulate its diagnostic utility or help confirm breast lineage in metastatic lesions (35). TMEM87A (transmembrane protein 87A), a recognized breast cancer immunohistochemistry staining target (36), displayed glycosylation (N62) and ubiquitination (K496). Finally, cathepsin D, a lysosomal protease linked to metastasis and hormonal therapy evasion (37, 38), was found to carry glycosylation (N263) and acetylation (K127). Together, these findings suggest that the unique combination of modifications may reveal new molecular mechanisms and serve as valuable targets for personalized therapeutic strategies.

## Discussion and Conclusion

The idea of integration was introduced in the development of this novel procedure to bridge the longstanding gaps in multi-PTM proteomics studies, which made possible the simultaneous quantitative analysis of phosphorylation, glycosylation, acetylation, and ubiquitination from the same sample (Fig. 1). Alternative methods for achieving this objective were systematically compared, establishing our workflow as a superior approach (Fig. 2, Fig. 3). Beyond streamlining workflows and reducing instrument time, our workflow demonstrated high sensitivity and specificity for the identification and quantification of modification-specific peptides and allowed for the discovery of previously unidentified modifications (Supplemental Fig. S3) and their interprotein/intraprotein crosstalks. Application of this workflow to breast cancer PDX models demonstrated a great potential for use in the identification of disease-associated changes in PTMs and for gaining insights into the complex regulatory networks that govern cellular processes (Fig. 4). This method is also highly flexible in terms of PTM selection and data acquisition scheme: more PTMs that utilize the affinity reaction could be included for added dimension of multi-PTM studies, and data acquisition scheme could be DDA, DIA, or hybrid.

By cross-validating the data generated by two orthogonal data acquisition platforms, our workflow achieves robust identification and quantification of PTM-specific peptides with unparalleled confidence (Fig. 2, Fig. 4, and Supplemental Fig. S2). The observed alignment in quantitative outcomes between DIA and TMT-based methods highlights the precision, reproducibility, and reliability of combining these two data acquisition platforms. The convergence of results provides a unified perspective on the intricate dynamics of PTMs, offering robust validation of findings across datasets. In addition, the consistent identifications observed through crossreferencing TMT–DDA and LFQ–DIA data lend greater credibility to the analysis compared with single-modality acquisition, reducing potential biases and enhancing confidence in the results. These findings establish our multi-PTM pipeline, with the integration of DIA, as a versatile and powerful tool for multi-PTM proteomics, facilitating high-resolution studies with unmatched accuracy and depth.

Another consideration for multi-PTM workflows is the order of sequential enrichments. In MoSAIC, we adopted the sequence IMAC (phosphopeptides) → MAX (IGPs) → IAP (acetylated and ubiquitinated peptides). This order was chosen primarily for buffer compatibility to minimize sample loss during buffer exchanges and to reduce matrix complexity before antibody-based capture. Although alternative orders (*e*.*g*., IAP-first) are technically feasible with additional buffer exchange steps, they add handling complexity and may decrease yield. Importantly, our variability analysis (Fig. 3*B*) did not show an increase in variability for later enrichments, supporting the robustness of the chosen sequence.

Scalability and adaptability are also hallmarks of this workflow. While we focused on four PTMs, antibody-based enrichment steps are modular—other modifications, such as phosphotyrosine, could be incorporated by adding compatible antibodies. However, expanding this workflow to incorporate additional PTMs warrants further investigation. The inclusion of more PTMs could have variable effects on the confident identification and quantification of modification-specific peptides because of the ion suppression effects (high-abundance peptides suppressing the ionization of low-abundance peptides) and signal competitions (resource allocation during data acquisition). Specifically, introducing more PTMs increases the diversity and number of peptide species entering the LC–MS/MS analysis, thereby raising the probability that peptides carrying different modifications (*e*.*g*., a phosphopeptide and an acetylated peptide with similar *m/z* and RT will be coisolated and fragmented together. In parallel, each enrichment chemistry has its own specificity profile and can introduce non-target peptides into the final multiplexed peptide mixture. These non-target species can compete with PTM peptides for ionization and instrument time. Another source of complexity could arise from incomplete TMT labeling, generating different peptide forms and diluting informative signal. Together, these intricacies highlight the need for systematic considerations when extending the workflow to include additional PTMs.

In MoSAIC, we mitigate these sources of interference by combining sequential, high-specificity PTM enrichments, offline fractionation to reduce sample complexity per fraction, and consistently high TMT labeling efficiency so that peptides are present predominantly as single, fully labeled species. Within this framework, enrichment specificity and fractionation primarily reduce co-isolation of cross-PTM and unmodified peptides, whereas high TMT labeling efficiency minimizes “self-interference” arising from mixed labeling states of the same peptide. In practice, optimization can be achieved by jointly tuning several orthogonal parameters, including the number of PTMs profiled, the extent of offline fractionation, the LC gradient length, and MS acquisition settings, such as precursor isolation window width, resolution, AGC target, maximum injection time, and dynamic exclusion. More extensive fractionation and/or longer gradients can be used when maximal depth and dynamic range are required for highly complex, multi-PTM samples, whereas modest fractionation combined with shorter gradients (*e*.*g*., ∼60 min) and appropriately tuned acquisition parameters can substantially increase throughput for applications with lower depth requirements or simpler sample matrices.

In conclusion, our integrated multi-PTM proteomics workflow MoSAIC offers a robust, sensitive, and flexible approach for the simultaneous analysis of multiple PTMs from the same sample. This methodology holds great promise for advancing our understanding of complex proteomic landscapes, identifying novel disease biomarkers, and uncovering potential drug targets. Future research should focus on optimizing the incorporation of additional PTMs and refining fractionation techniques to further enhance the capabilities and applications of this innovative approach.

## Resource Availability

### Lead Contact

Further information and requests for resources should be directed to and will be fulfilled by the lead contact, Hui Zhang (huizhang@jhu.edu).

### Material Availability

This article contains no unique reagents or resources. All antibodies and reagents are available commercially.

### Data and Code Availability

The MS raw data (.raw), peak list files (.mzML), processed search results (.mzid), and Spectronaut project files (.sne) have been deposited in the ProteomeXchange Consortium *via* the PRIDE repository under the dataset identifier PXD061601. Additional information required to reanalyze the data reported in this study is available from the Lead Contact upon reasonable request.

## Supplemental Data

This article contains supplemental data.

## Conflict of Interest

The authors declare no competing interests.

## Figures and Tables

**Supplemental Figure S1A:**
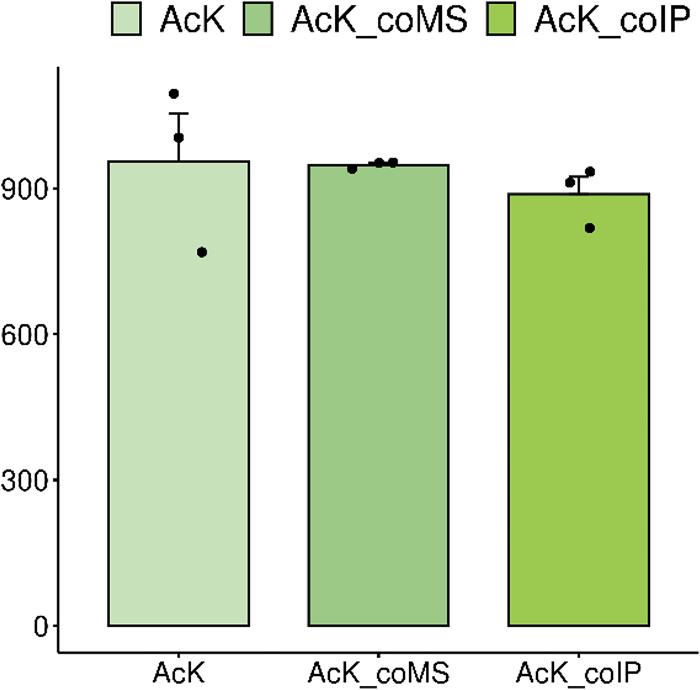
Label-free identifications of acetylated peptides from individual enrichments, coMS and coIP.

**Supplemental Figure S1B:**
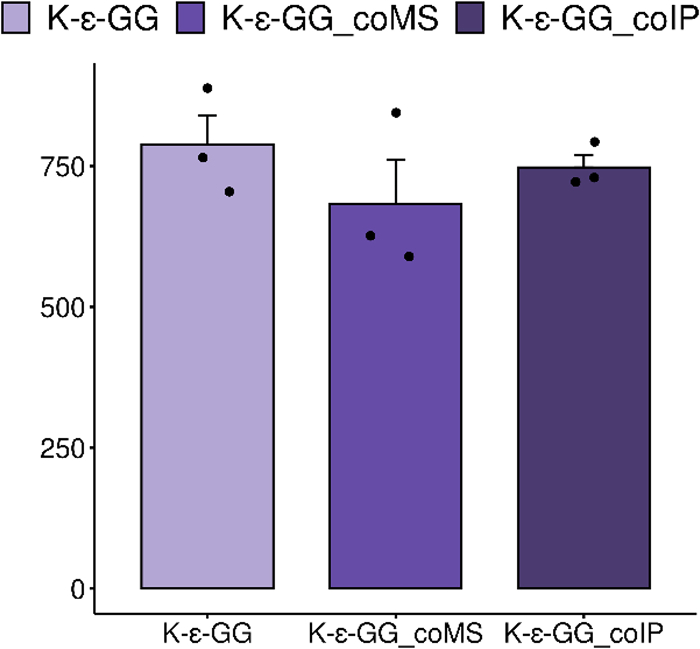
Label-free identifications of ubiquitinated peptides from individual enrichments, coMS and coIP.
